# Read My Face: Automatic Facial Coding Versus Psychophysiological Indicators of Emotional Valence and Arousal

**DOI:** 10.3389/fpsyg.2020.01388

**Published:** 2020-06-19

**Authors:** T. Tim A. Höfling, Antje B. M. Gerdes, Ulrich Föhl, Georg W. Alpers

**Affiliations:** ^1^Department of Psychology, School of Social Sciences, University of Mannheim, Mannheim, Germany; ^2^Business Unit, Pforzheim University of Applied Sciences, Pforzheim, Germany

**Keywords:** automatic facial coding, facial electromyography, skin conductance, emotion, facial expression

## Abstract

Facial expressions provide insight into a person’s emotional experience. To automatically decode these expressions has been made possible by tremendous progress in the field of computer vision. Researchers are now able to decode emotional facial expressions with impressive accuracy in standardized images of prototypical basic emotions. We tested the sensitivity of a well-established automatic facial coding software program to detect spontaneous emotional reactions in individuals responding to emotional pictures. We compared automatically generated scores for valence and arousal of the Facereader (FR; Noldus Information Technology) with the current psychophysiological gold standard of measuring emotional valence (Facial Electromyography, EMG) and arousal (Skin Conductance, SC). We recorded physiological and behavioral measurements of 43 healthy participants while they looked at pleasant, unpleasant, or neutral scenes. When viewing pleasant pictures, FR Valence and EMG were both comparably sensitive. However, for unpleasant pictures, FR Valence showed an expected negative shift, but the signal differentiated not well between responses to neutral and unpleasant stimuli, that were distinguishable with EMG. Furthermore, FR Arousal values had a stronger correlation with self-reported valence than with arousal while SC was sensitive and specifically associated with self-reported arousal. This is the first study to systematically compare FR measurement of spontaneous emotional reactions to standardized emotional images with established psychophysiological measurement tools. This novel technology has yet to make strides to surpass the sensitivity of established psychophysiological measures. However, it provides a promising new measurement technique for non-contact assessment of emotional responses.

## Introduction

Emotions motivate to approach rewards or avoid punishments and they play a critical role in everyday human social interaction. Emotional facial expression is a core aspect of emotion processing in humans (Scherer and Ellgring, 2007; Keltner and Cordaro, 2017; Sander et al., 2018; Scherer and Moors, 2019). Thus, detection of facial expression might give an insight into one’s emotional processing. In order to measure emotional facial expressions, researchers typically use either certain observation techniques or record the activity of specific muscles with facial electromyography (EMG; Mauss and Robinson, 2009; Wolf, 2015). Observation techniques are typically based on the Facial Action Coding System (FACS; Ekman et al., 2002), for which the observable activity of specific muscle groups are labeled and coded as action units (AU) by human coders. Relevant AUs involved in basic emotion facial expression are identified in this framework (EMFACS; Ekman et al., 1994).

Recent advances in technology have enabled emotion researchers to obtain AU activity and consecutive emotion measurements automatically through analysis of video and photo recordings (Pantic and Rothkrantz, 2000; Cohn and Sayette, 2010). Compared to human observation, automatic facial coding is less time consuming and always blind to the research hypothesis (for an overview of analysis systems see Poria et al., 2017). Even in comparison to electrode-based measures, it is less invasive and less susceptible to motion artifacts (Schulte-Mecklenbeck et al., 2017). Furthermore, video-based measurements do not require preparation or application of electrodes and hence are more flexible for data collection (e.g., online research). For these reasons, automatic facial coding may be the preferable measurement technique to detect emotional facial responses in a broad spectrum of research fields.

### Automatic Facial Coding

Converging evidence shows that automatic facial coding (AFC) provides sensitive and specific scores for emotional intensities, as well as associated AUs, in highly standardized and prototypical facial expression inventories for static photographs (Bijlstra and Dotsch, 2011; Mavadati et al., 2013; Lewinski et al., 2014; Lewinski, 2015) and dynamic videos (Calvo et al., 2018). Furthermore, these findings can also be generalized to tasks where facial expressions are mimicked by real persons (Stöckli et al., 2018; Beringer et al., 2019; Sato et al., 2019). Summarizing these results, pleasant facial expressions (happy) are detected with higher probabilities compared to unpleasant facial expressions (anger, sadness, disgust, or anxiety) and misattributions of specific emotions (e.g., surprise in scared faces) can be observed. Furthermore, AFC of mimicked pleasant and unpleasant facial expressions correlate strongly with EMG measurements within the same participants (Beringer et al., 2019). However, these detection patterns are typically even stronger pronounced in untrained human observers (Nummenmaa and Calvo, 2015; Calvo and Nummenmaa, 2016).

Findings indicate that AFC is a suitable measurement alternative to human observers, in particular if recordings are made under optimal conditions (e.g., lighting, face angle, no speech, and no face coverage) and the facial expression shows intense prototypical basic emotion configurations. Photos and videos of well-trained actors, showing specific emotions in an exaggerated, FACS-coordinated manner are indeed useful for basic testing of the measuring systems. However, they do not necessarily reflect naturally occurring emotional facial reactions. The use of such validation material can be informative in terms of the upper limit performance for these six basic emotions, but may not be suitable for testing the sensitivity of detecting spontaneously occurring emotional responses. Although this is a necessary first step, it does not yet prove that measurement sensitivity is sufficient for spontaneously and naturally occurring emotional expressions (e.g., Cacioppo et al., 2000; Matsumoto et al., 2008) and yet has rarely been proven to be an ecological valid measurement tool.

Only two studies elicited actively emotional facial responses in human participants/observers and analyzed them with a computer vision approach. In one preliminary study only a small set of pictures – three pleasant and unpleasant emotional scenes – were used to elicit facial responses with moderate to good classification performance on a categorical analysis level (Stöckli et al., 2018). The other study demonstrated good prediction of unpleasant versus pleasant facial responses with an AU-based machine learning procedure (Haines et al., 2019). Unfortunately, in both studies there was no neutral picture category as a comparative condition.

### Valence and Arousal in Psycho-Physiological Research

In providing scores for valence and arousal, the FR follows psychological models of emotion that highlight the importance of a two-dimensional affective space (Russell, 1980; Russell and Barrett, 1999; Barrett and Bliss-Moreau, 2009; but there are other models that include additional dimensions, e.g., Fontaine et al., 2007; Bakker et al., 2014). Valence ranges from pleasant to unpleasant, whereas the arousal dimension ranges from not arousing to highly arousing emotional states. In turn, these dimensions usually elicit approach and withdrawal behavior or behavioral tendencies, and activate the corresponding motor preparedness (Davidson, 1992; Bradley et al., 2001; Lang and Davis, 2006). Valence and arousal are thought to portray primarily independent processes, in that arousal does not simply correspond to the intensity of a current pleasant or unpleasant affective state (Kuppens et al., 2013). Additionally, there is evidence that specific neural structures are involved in processing pleasant and unpleasant arousal levels (Gerdes et al., 2010). Facial reactions are known to mirror valence evaluations and occur unintentionally in the presence of emotional stimuli (Neumann et al., 2005; Eisenbarth et al., 2011), even if they are processed near-threshold (Neumann et al., 2014). Valence-type reactions are indicated by facial reactions and changes in autonomic activity, such as variations to sweat glands or heart rate, which are associated with arousal processes (Siegel et al., 2018). However, enhanced arousal levels modulate the intensity of facial reactions (Fujimura et al., 2010).

EMG of the corrugator and zygomaticus muscles is frequently used to measure the processing of emotion (Cacioppo et al., 2000; Larsen et al., 2003; Huang et al., 2004; Tassinary et al., 2007; Reisenzein et al., 2013). The corrugator is related linearly with the self-reporting of hedonic valence, manifesting in an increase of activity for unpleasant emotions and a decrease for pleasant emotional states (Hess and Blairy, 2001; Rymarczyk et al., 2011). In particular, corrugator activity distinguishes strongly between different pleasant and unpleasant facial expressions (Wolf et al., 2005). The zygomaticus on the other hand is selectively activated in pleasant states elicited by emotional images (Lang et al., 1993; Sato et al., 2008; Baur et al., 2015).

There are notable differences in the rationale of AFC and EMG-measurements: While EMG, in particular, measurements of the corrugator and the zygomaticus muscles, are expected to correlate with the core affective dimension of valence, AFC is typically trained to recognize intensities of basic emotional facial expressions. Correspondingly, the valence parameter generated by AFC is also grounded in this logic. However, the basic emotion approach can also be projected in the core affect framework (Posner et al., 2005; Panksepp, 2007; Yik et al., 2011).

Research regarding indicators of emotional arousal focuses on peripheral physiological measurements. A recent meta-analysis (Siegel et al., 2018) compared different physiological measures such as sweat gland activity, cardiovascular activity, respiration, and body temperature; these are often used in emotion research. In general, physiological indicators are more highly modulated by emotional compared to neutral stimuli. Skin Conductance (SC) in particular is not a very specific measure for different basic emotions, as increases in SC activity are induced by multiple emotional states (Kreibig, 2010). However, SC is a highly sensitive measure of emotional arousal compared to respiration or heart rate (Mendes, 2009). SC also correlates strongly with verbal reports of arousal during the viewing of emotional pictures (Lang et al., 1993). Furthermore, SC shows high coherence to continuous self-reports of emotional arousal elicited by dynamic emotional videos (Golland et al., 2014). Emotional arousal measured by SC increases while viewing high arousing images, both pleasant and unpleasant, compared to low arousing or neutral pictures (Bradley et al., 2008; Costa and Esteves, 2008).

### Research Questions

While standardized inventories provide a clear-cut norm for the evaluation of AFC (i.e., the emotion categories of the inventory), the measurement of spontaneous expressions would require an external criterion. Importantly, previous studies have used test material (e.g., standardized pictures), that are similar to the software’s training material. Hence, we argue that a critical standard would be to test FR against other well-established psychophysiological indicators of emotion like EMG and SC. In order to use FR to score natural expressions, a test under more naturalistic conditions is needed. The presented study directly compares the measurement performance of FR indicators of emotional expressions from human participants with measurements from physiological channels in a naturalistic setting. This, however, has not yet been attempted so we set out to close this research gap. In order to induce emotional expressions in our participants, standardized emotion-eliciting pictures were presented in a typical free viewing paradigm. This will provide essential information on the (relative) usefulness of AFC in emotion research.

Thus, we used the different measures to analyze spontaneous emotional reactions to pleasant, unpleasant and neutral images varying in arousal from the International Affective Picture System (IAPS; Lang et al., 2008) were analyzed in order to compare the different measures. Furthermore, valence measures provided by FR were compared to changes in facial EMG. We hypothesized that both measures differ between responses to pleasant, neutral, and unpleasant stimuli as a function of emotional valence. In addition, we tested the hypothesis that overall facial movement – i.e., arousal measures provided by FR – reflects an emotional arousal component. The electrode-based equivalent for the FR Arousal measure was SC. We hypothesize that both measures show elevated signals for arousing (pleasant and unpleasant) compared to neutral pictures. The relationships between measurement sensitivity, specificity indicators and self-report ratings were assessed. In general, it has been shown that EMG and SC are both highly sensitive indicators of emotional valence and arousal (e.g., Haag et al., 2004). Hence, it is expected that both electrode-based measures correlate substantially and specifically with the corresponding self-report dimension. Concerning FR measures, a similar association pattern should be observed if video-based measures perform as sensitively and specifically as established psychophysiological emotion measurement procedures. Accordingly, FR measures of valence and arousal are thought to correlate sensitively and specifically with corresponding self-report of valence and arousal.

## Materials and Methods

### Participants

A total of 43 volunteers (14 males) participated in the experiment. Age varied between 19 and 50 years (*M* = 23.21, *SD* = 5.30)^1^. Eight participants were left-handed. Ethnicity was mostly European, with three participants of African descent, one of Asian descent, and two from the Middle East. General exclusion criteria included being under 18 years of age, use of psychoactive medication, acute episode of mental disorders, or severe somatic diseases, as well as those who have a beard or wear glasses. Three participants were excluded prior to the analyses due to computer failures. Participants with corrected vision were asked to wear contact lenses during the experiment. All participants were students of the University of Mannheim and received either 8€ compensation or course credit for participation. Furthermore, all participants signed informed consent before the data collection. The experiment was approved by University Research Ethics Committee.

### Questionnaires

A socio-demographic questionnaire (e.g., gender, age, educational level), the Social Interaction Anxiety Scale (SIAS; Stangier et al., 1999), the State-Trait Anxiety Inventory (STAIstate and STAItrait; Laux et al., 1981), the Positive-and-Negative-Affect-Schedule (PANASpos and PANASneg; Krohne et al., 1996), the Self-Rating Depression Scale (SDS; Zung, 1965) as well as the Berkley Expressivity Questionnaire (BEQ; Mohiyeddini et al., 2008) were administered before starting the main experiment. Scores of the questionnaires were in the normal range regarding SDS (*M* = 36.09, *SD* = 8.39, *Cronbachs α* = 0.84), STAIstate (*M* = 39.44, *SD* = 7.24, *Cronbachs α* = 0.85), STAItrait (*M* = 41.56, *SD* = 9.19, *Cronbachs α* = 0.90), BEQ (*M* = 24.43, *SD* = 3.86, *Cronbachs α* = 0.86), PANASpos (*M* = 30.00, *SD* = 6.35, *Cronbachs α* = 0.85), and PANASneg (*M* = 13.53, *SD* = 4.33, *Cronbachs α* = 0.85). However, the sample has slightly elevated scores from the average on the SIAS (*M* = 20.77, *SD* = 12.22, *Cronbachs α* = 0.89), which is a common observation in student samples.

### Stimulus Material

Sixty pictures were selected from the International Affective Picture System^2^ (IAPS; Lang et al., 2008) consisting of 24 pleasant arousing (animals or babies, landscapes, erotica couples, erotica solo), 24 unpleasant arousing (grief, pollution, human attacks, mutilations), and 12 neutral non-arousing scenes (household objects, neutral human). Each of the 10 groups of pictures were represented by 6 IAPS scenes. Because neutral scenes typically induce less variable responses, fewer pictures were selected for this category. The rational for scene selection was two-fold: First, pleasant, neutral, and unpleasant scenes should clearly differ in valence. Second, pleasant and unpleasant scenes should not differ in arousal, but should have higher arousal levels than neutral scenes. Based on averaged IAPS database ratings, the stimulus categories varied strongly in terms of valence, *F*(2,57) = 766.07, *p* < 0.001, η^2^ = 0.96, and arousal, *F*(2,57) = 23.89, *p* < 0.001, η^2^ = 0.46. Pleasant scenes were rated as more positive, *M* = 6.90, *SD* = 0.48, *t*(34) = 12.94, *p* < 0.001, *d* = 5.13, and unpleasant scenes were rated as more negative, *M* = 2.38, *SD* = 0.39; *t*(34) = 22.20, *p* < 0.001, *d* = 8.76, compared to neutral scenes, *M* = 5.04, *SD* = 0.18. Pleasant scenes, *M* = 5.24, *SD* = 1.20, *t*(34) = 6.07, *p* < 0.001, *d* = 2.35, and unpleasant scenes, *M* = 5.60, *SD* = 1.18, *t*(34) = 7.16, *p* < 0.001, *d* = 2.77, were rated as more arousing compared to neutral scenes, *M* = 3.00, *SD* = 0.61, and had comparable arousal levels, *t*(46) = 1.04, *p* = 0.796, *d* = 0.30, according to IAPS rating database.

### Procedure

**FIGURE 1:**
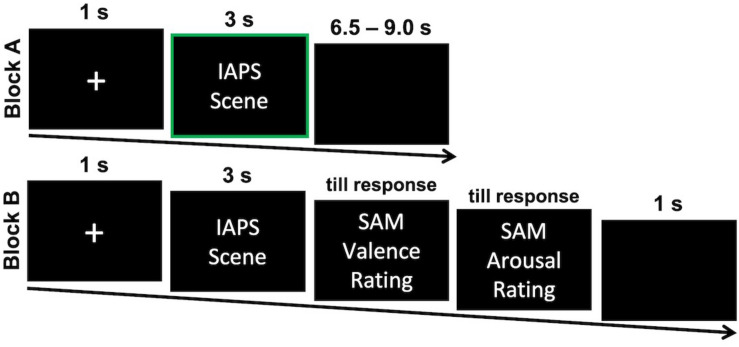
One exemplary trail for each of the two experimental blocks. Participants attentively viewed the presented IAPS scenes in Block A first and responded to self-report scales for each scene Block B afterward. EMG, SC, and FR measurements were analyzed in response to the presentation in Block A as indicated by a green frame. IAPS, International Affective Picture System; SAM = Self-Assessment Manikin.

Following informed consent and completion of the questionnaires, participants used a medical skin exfoliant on areas of their faces in order to improve EMG measurement signal where electrodes were next attached. Participants were told to make a neutral facial expression for 10 s at the beginning of the experiment. This time interval served as individual calibration period for FR measurements. The experimental trials were presented in two subsequent blocks (see Figure 1 for an illustration). In order to familiarize participants with the specific task, 5 practice trials preceded both blocks. In the first block participants were instructed to “attentively view the presented scenes.” Each picture was indicated by a 1 s fixation cross, presented for 3 s, and followed by an inter-trial-interval with pseudorandomized durations between 6500 and 9000 ms, with a mean of 7750 ms, to avoid habituation. Presentation order was randomized such that a maximum of three pictures from the emotion *stimulus category* were shown in a row to avoid habituation effects. After the first block, a short break was incorporated before block two started. Afterward, the participants were asked to evaluate the pictures. The 60 pictures were shown in the exact same order for 3 s and were immediately followed by two visual rating scales (Bradley and Lang, 1994). Participants rated how they felt during picture presentation regarding emotional valence (1 = very unpleasant, 5 = neutral, 9 = very pleasant) and emotional arousal (1 = not at all aroused, 9 = very aroused). Both scales were inverted to improve interpretability.

### Apparatus and Measurements Preprocessing

High-precision software (E-Prime; Version 2.0.10; Psychology Software Tools) was used for picture presentation. Pictures were shown centrally on a 19-inch monitor with a resolution of 1024 × 768 approximately 70 cm away from the participant. Optimal illumination with diffused frontal light was maintained throughout. EMG and SC were measured in a bipolar fashion with reusable Ag/AgCl electrodes that had a contact surface diameter of 5 mm. EMG electrodes were placed on the zygomaticus major and corrugator supercilii on the left facial hemisphere, following the recommendations of Fridlund and Cacioppo (1986). SC electrodes were mounted on the left hand palm. Electrodes were filled with isotonic gel. EMG and SC activity was recorded with 1000 Hz sampling rate using Brainvision amplifier and recording system (V-Amp Edition; Version 1.20.0601). EMG signals were rectified then integrated with a time constant of 5.3 ms, as well as a high (250 Hz), low cutoff (30 Hz), and notch (50 Hz) filter (Fridlund and Cacioppo, 1986). EMG measurements were analyzed combined as the difference between the mean activities of zygomaticus and the corrugator (EMG Delta). Positive values of this combined measure indicate activation of the zygomaticus and deactivation of the corrugator muscle and can be interpreted as pleasant valence measure. Conversely, negative values indicate activation of the corrugator and deactivation of the zygomaticus muscles and can be interpreted as an unpleasant valence measure. This rationale improved comparability between EMG measurements and video-based assessment of valence parameters (i.e., FR Valence). A separate analysis of corrugator and zygomaticus muscle activity is reported in Supplementary Material A. SC activities were measured and preprocessed following the recommendations of Boucsein et al. (2012). Signals were filtered using *Butterworth Zero Phase Filters* with a low cutoff of 0.0159 Hz, a high cutoff of 5 Hz, a notch filter of 50 Hz, and were additionally divided by −25 × 10^3^ to obtain micro Siemens as unit.

**FIGURE 2:**
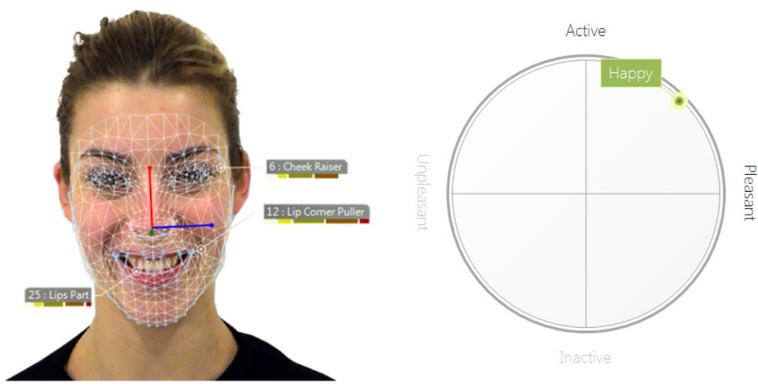
Example of the automatic facial coding analysis of the Facereader software (Noldus Information Technology). **(Left)** Depicted is a happy facial expression from the ADFES inventory (model F01; Van Der Schalk et al., 2011). The net represents the digital face model which establishes distance measures between distinct facial features. Based on this information, activity of specific action units is estimated. **(Right)** In a next step, the current profile of action unit activities is integrated to higher order emotion measures (in this case the basic emotion happiness, pleasant valence, and relatively high arousal).

Videos of participants’ faces were recorded with Logitec HDC 615 Webcamera, which was placed above the computer screen. Videos were processed using Facereader Software (FR; Version 7.0, Noldus Information Technology) and Observer XT (Version 12.5, Noldus Information Technology). The software’s artificial intelligence is trained to register activation of 20 AUs (i.e., 1, 2, 4, 5, 6, 7, 9, 10, 12, 14, 15, 17, 20, 23, 24, 25, 26, 27, and 43) and to indicate scores for happy, surprised, angry, sad, disgusted, and scared faces as proposed by the basic emotion framework (Ekman, 2017). The visual pattern classifier is based on deep learning networks to extract visual features from pictures or videos and calculate intensity estimations for each specific emotion. In accordance with neuro-computational models human face processing (Dailey et al., 2002; Calder and Young, 2005), FR detects facial configurations in the following steps (Van Kuilenburg et al., 2005, 2008): (1) The C*ascade classifier algorithm* finds the position of the face (Viola and Jones, 2004). (2) Face textures are normalized and the a*ctive appearance model* synthesizes a digital face model representing facial structure with over 500 location points (Cootes and Taylor, 2004). (3) Compressed distance information is then transmitted to an artificial neural network (Bishop, 2010). (4) Finally, the artificial neural network connects these scores with relevant emotional labels through supervised training with over 10.000 samples (pictures) of emotional faces, to classify relative intensity of a given facial configuration (see Figure 2 for an examples). On the most integrated level, FR provides scores for valence and arousal. FR software calculates FR Valence (pleasant to unpleasant) as the difference between pleasant and unpleasant emotion intensities. FR Arousal (inactive to active) is an index of overall AU activation^3^. FR measurements were calibrated per participant as recommended by the software manual. The East-Asian or elderly face-model was applied where appropriate instead of the general face-model. Originally, FR Valence scale ranged from −1 (unpleasant facial expression) to 1 (pleasant facial expression), and FR Arousal ranged from 0 (not active) to 1 (active). For better interpretability, both scales were multiplied by 100. All measures (EMG, SC, FR Valence, and FR Arousal) were baseline-corrected for each trial – i.e., mean activation of the second before stimulus onset (baseline) was subtracted from following stimulus modulated activity.

### Data Reduction and Analysis

The averages of psycho-physiological and video-based measurements as well as self-report ratings were calculated for all pictures of one *stimulus category* (pleasant, neutral, and unpleasant). To account for changes over time, activities were averaged in 1-s intervals for 5 s after stimulus onset. To assess effects on FR, EMG, and SC, 3 × 5 ANOVAs were calculated separately regarding the within-subjects factors *stimulus category* and *time window* (s1, s2, s3, s4, and s5). We applied Greenhouse and Geisser (1959) correction where appropriate. The 95% confidence intervals were estimated using 5000 bootstrap samples based on percentiles (Efron, 1987). Eta-squared (η^2^) was reported as effect size for *F*-Tests (Levine and Hullett, 2002) (η^2^ ≥ 0.01 small; η^2^ ≥ 0.06 medium; η^2^ ≥ 0.14 large; Pierce et al., 2004). Cohen’s *d* was reported for *t*-tests (*d* ≥ 0.2 small; *d* ≥ 0.5 medium; *d* ≥ 0.8 large; Cohen, 1988). Bonferroni-Correction for *p*-values was applied for all *post hoc t*-tests (α = 0.05/3). In addition to univariate analysis of the different measures, Pearson correlations between self-report ratings of valence and arousal, measures of FR, EMG, and SC were reported. All data was averaged per picture over participants and *z*-standardized for each physiological and behavioral measurement for their most active time windows (EMG: 1–3 s; SC, AFC: 3–5 s) so that all correlations would improve in comparability.

## Results

### Ratings of Valence and Arousal

Analysis of the emotional self-report scales showed the expected pattern for valence and arousal rating of the stimulus material (see Table 1)^4^. The ANOVA for the valence ratings revealed strong differences between *stimulus categories*, *F*(2,84) = 467.94, *p* < 0.001, η^2^ = 0.92. *Post hoc* comparison showed that pleasant pictures were rated to be more pleasant than unpleasant pictures, *t*(42) = 23.56, *p* < 0.001, *d* = 3.59, or neutral pictures, *t*(42) = 14.59, *p* < 0.001, *d* = 2.22. Correspondingly unpleasant pictures were rated as more unpleasant than neutral pictures, *t*(42) = 22.37, *p* < 0.001, *d* = 3.41. The arousal ratings also showed a strong effect of *stimulus categories*, *F*(2,84) = 152.21, *p* < 0.001, η^2^ = 0.78. Pleasant pictures, *t*(42) = 14.29, *p* < 0.001, *d* = 2.18, as well as unpleasant pictures, *t*(42) = 15.30, *p* < 0.001, *d* = 2.33, were rated as more arousing than neutral pictures. Unpleasant pictures were rated more arousing than pleasant pictures, *t*(42) = 5.35, *p* < 0.001, *d* = 0.82, respectively.

**TABLE 1:** Mean valence and arousal ratings (standard deviation in parenthesis, 95% confidence intervals in square brackets) of the picture categories.

	Pleasant	Neutral	Unpleasant
Valence ratings	6.61 (0.72) [6.40; 6.82]	5.02 (0.26) [4.94; 5.09]	2.51 (0.72) [2.31; 2.73]
Arousal ratings	4.56 (1.19) [4.22; 4;90]	2.04 (1.03) [1.75; 2.36]	5.79 (1.41) [5.36; 6.19]

### Valence Measurements

#### Facereader (FR) Valence

**FIGURE 3:**
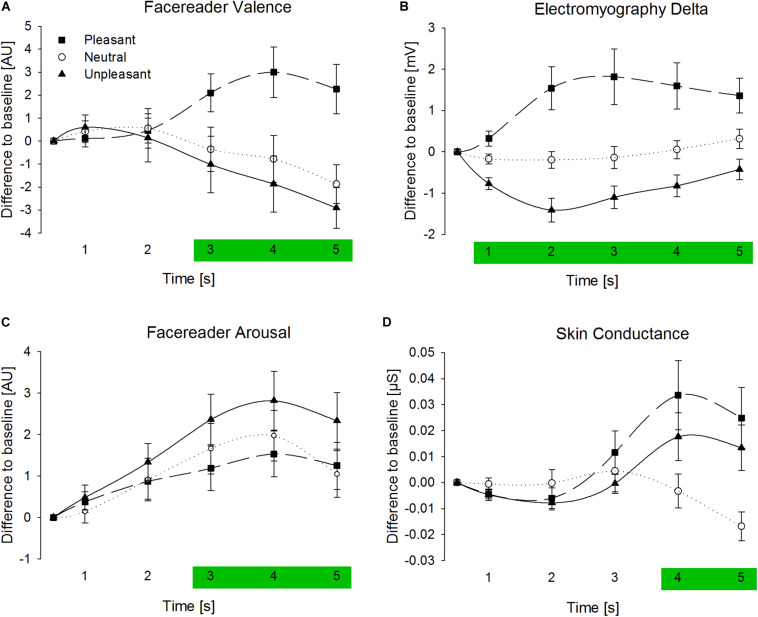
Averaged signals of Facereader Valence **(A)** Electromyography Delta **(B)**, Facereader Arousal **(C)**, and Skin Conductance **(D)** separate for time windows in seconds after picture onset (three seconds stimulus presentation) and stimulus category (difference to baseline in Arbitrary Units [AU], millivolt [mV], or micro Siemens [μS]). Error bars are standard errors of the mean. Green areas highlight time windows with significant stimulus category effects.

Analysis of FR Valence revealed a strong interaction between *stimulus category* and *time window*, *F*(8,336) = 10.89, *p* < 0.001, η^2^ = 0.21, followed by a significant main-effect for *stimulus category*, *F*(2,84) = 5.72, *p* = 0.006, η^2^ = 0.12, and no significant main effect for *time window*, *F*(4,168) = 1.15, *p* = 0.321, η^2^ = 0.03 [see Supplementary Material A for additional analysis of AU4 (Brow Lowerer) and AU12 (Lip Corner Pull)]^5^. Hence, effects of *stimulus category* were analyzed separately for each *time window* (see Table 2A). Separate analyses of the *stimulus category* for the *time windows* revealed no effects for s1 and s2 (*p* > 0.10), and highly significant effects for s3, *F*(2,84) = 5.76, *p* = 0.006, η^2^ = 0.12, s4, *F*(2,84) = 9.05, *p* < 0.001, η^2^ = 0.18, and s5, *F*(2,84) = 9.40, *p* < 0.001, η^2^ = 0.18, after stimulus onset (see also Figure 3A). *Post hoc* comparison between *stimulus categories* of the s3–s5 showed that measures were significantly more positive for pleasant pictures compared to neutral, *p* = [0.017; 0.003], *d* = [0.44; 0.54], or unpleasant pictures, *p* = [0.017; 0.001], *d* = [0.45; 0.60]. However, FR Valence did not differ between neutral and unpleasant pictures, *p* = 1.00, *d* = [0.10; 0.14]. Overall, FR Valence detected moderate differences between responses to pleasant and unpleasant or between pleasant and neutral pictures. No differences between reactions to neutral and unpleasant pictures can be reported, which might indicate a lowered sensitivity of FR Valence in the detection of unpleasant facial expression or a negative trend for neutral responses. Explorative comparison of FR Valence against the baseline (i.e., zero) showed significant differences for pleasant stimulus material peaking in s4, *t*(42) = 2.73, *p* = 0.009, *d* = 0.42, and for unpleasant stimulus material peaking in s5, *t*(42) = 3.28, *p* = 0.002, *d* = 0.50. However, in s5 neutral pictures led also to an negative trend in FR Valence, *t*(42) = 2.22, *p* = 0.032, *d* = 0.34, which favors the interpretation that FR Valence shows a negative trend for neutral stimulus material.

**TABLE 2:** Mean Facereader valence (2A) and electromyography delta (2B, standard deviations in parenthesis, 95% confidence intervals in square brackets, difference to baseline in arbitrary units [AU] or millivolt [mV]), separately for time windows and stimulus categories.

	(2A) Facereader Valence [AU]					
	
	Pleasant	Neutral	Unpleasant	*F* (2,84)	*p*-value	η^2^
1st second	0.11 (2.39) [−0.58; 0.83]	0.41 (3.15) [−0.46; 1.38]	0.60 (3.55) [−0.29; 1.77]	0.63	0.536	0.02
2nd second	0.46 (3.58) [−0.59; 1.52]	0.55 (5.62) [−1.12; 2.20]	0.14 (6.82) [−1.77; 2.32]	0.20	0.200	0.01
3rd second	2.10 (5.42) [0.55; 3.79]	−0.36 (6.28) [−2.26; 1.43]	−1.02 (8.04) [−3.33; 1.51]	5.76	0.005	0.12
4th second	3.00 (7.20) [0.96; 5.28]	−0.78 (6.73) [−2.80; 1.20]	−1.87 (7.97) [−4.08; 0.69]	9.05	<0.001	0.18
5th second	2.26 (7.08) [0.30; 4.56]	−1.87 (5.52) [−3.51; −0.22]	−2.91 (5.81) [−4.57; −1.14]	9.40	<0.001	0.18

	**(2B) Electromyography Delta [mV]**					
	
	**Pleasant**	**Neutral**	**Unpleasant**	***F* (2,84)**	***p*-value**	**η^2^**

1st second	0.32 (1.20) [0.00; 0.71]	−0.17 (0.81) [−0.40; 0.07]	−0.77 (0.91) [−1.05; −0.51]	21.83	<0.001	0.34
2nd second	1.54 (3.42) [0.64; 2.65]	−0.19 (1.31) [−0.57; 0.19]	−1.41 (1.87) [−1.97; −0.88]	17.10	<0.001	0.29
3rd second	1.81 (4.43) [0.64; 3.24]	−0.14 (1.74) [−0.65; 0.39]	−1.10 (1.80) [−1.67; −0.58]	13.44	<0.001	0.24
4th second	1.59 (3.69) [0.58; 2.75]	0.05 (1.43) [−0.38; 0.47]	−0.82 (1.76) [−1.37; −0.33]	12.02	<0.001	0.22
5th second	1.34 (2.75) [0.60; 2.23]	0.31 (1.52) [−0.12; 0.77]	−0.42 (1.62) [−0.92; 0.04]	8.92	<0.001	0.18

*Statistics correspond to ANOVA effects of the stimulus category.*

#### Electromyography (EMG) Delta

The ANOVA for EMG Delta showed a strong interaction between *stimulus category* and *time window*, *F*(8,336) = 6.61, *p* = 0.001, η^2^ = 0.14, a strong main effect for *stimulus category*, *F*(2,84) = 15.14, *p* < 0.001, η^2^ = 0.27, and a moderate effect for *time window*, *F*(4,168) = 2.73, *p* = 0.082, η^2^ = 0.06 (see Supplementary Material A for separate analysis of corrugator and zygomaticus)^5^. Hence, effects of *stimulus category* were analyzed separately for each *time window* (see also Table 2B). *Stimulus categories* strongly modulated EMG Delta activities during picture presentation, *F*(2,84) = [8.92; 21.83], *p* < 0.001, η^2^ = [0.18; 0.34], immediately after stimulus onset (see also Figure 3B). *Post hoc* comparison for these t*ime windows* showed that compared to neutral pictures, values were more positive for pleasant, *p* = [0.003; 0.002], *d* = [0.53; 0.56], and more negative for unpleasant pictures, *p* = [0.052; 0.002], *d* = [0.38; 0.56]. EMG response differentiated rather strongly between pleasant and unpleasant stimulus material, *p* ≤ 0.001, *d* = [0.62; 0.86]. Taken together, EMG signals differentiated between all picture categories and varied rather strongly between pleasant and unpleasant pictures.

#### FR Valence Versus EMG Delta

Comparing the strongest effects of EMG Delta and FR Valence showed comparable differences between response to pleasant and neutral pictures, *d*_*EMG*_ = 0.56 vs. *d*_*FR*_ = 0.54. However, only EMG Delta showed a substantial difference between neutral and unpleasant stimulus material, *d*_*EMG*_ = 0.56 vs. *d*_*FR*_ = 0.14. Furthermore, EMG signals differed between picture categories immediately after stimulus presentation, whereas FR Valence showed an unexpected long latency of 2 s.

### Arousal Measurements

#### Facereader (FR) Arousal

Regarding FR Arousal measures, a marginal significant interaction-effect between *stimulus category* and *time*, *F*(8,336) = 2.27, *p* = 0.091, η^2^ = 0.05, a significant main-effect for *stimulus category*, *F*(4,84) = 3.89, *p* = 0.030, η^2^ = 0.09, and a strong and significant main-effect for *time* can be reported, *F*(4,168) = 8.60, *p* = 0.001, η^2^ = 0.17^5^. In order to detect time-dependent effects of the *stimulus categories*, *time windows* are analyzed separately (see Table 3A). In accordance with FR Valence, FR Arousal measures also showed no effects for *stimulus category* for the first 2 s after stimulus onset (*p* > 0.10), and had moderate effects during s3, *F*(2,84) = 3.77, *p* = 0.031, η^2^ = 0.08, s4, *F*(2,84) = 3.82, *p* = 0.033, η^2^ = 0.08, and s5, *F*(2,84) = 3.28, *p* = 0.049, η^2^ = 0.07. *Post hoc* comparisons for these *time windows* revealed exclusively significant effects between response to pleasant and unpleasant stimulus material, *p* = [0.090; 0.027], *d* = [0.45; 0.60]. Thus, unpleasant compared to pleasant emotional scenes elicited stronger overall movement in the face indicated by FR Arousal (see also Figure 3C). No other pair-wise comparison of *stimulus categories* reached significance, *p* > 0.10.

**TABLE 3:** Mean Facereader arousal and skin conductance (standard deviations in parenthesis, 95% confidence intervals in square brackets, difference to baseline in arbitrary units [AU] or microsiemens [μS]), separately for time windows and stimulus categories.

	(3A) Facereader Arousal [AU]					
	
	Pleasant	Neutral	Unpleasant	*F* (2,84)	*p*-value	η^2^
1st second	0.37 (1.63) [−0.04; 0.88]	0.14 (1.78) [−0.32; 0.70]	0.48 (1.96) [−0.01; 1.10]	2.30	0.111	0.05
2nd second	0.87 (2.81) [0.14; 1.74]	0.91 (3.36) [0.04; 1.98]	1.33 (2.95) [0.55; 2.26]	1.37	0.261	0.03
3rd second	1.19 (3.55) [0.22; 2.28]	1.66 (4.00) [0.59; 2.89]	2.36 (3.98) [1.28; 3.49]	3.77	0.031	0.08
4th second	1.53 (3.62) [0.54; 2.62]	1.97 (4.00) [0.87; 3.17]	2.81 (4.64) [1.56; 4.27]	3.82	0.033	0.08
5th second	1.25 (3.72) [0.25; 2.36]	1.05 (3.70) [0.02; 2.17]	2.33 (4.47) [1.13; 3.74]	3.28	0.049	0.07

	**(3B) Skin Conductance [μS]**					
	
	**Pleasant**	**Neutral**	**Unpleasant**	***F* (2,84)**	***p*-value**	**η^2^**

1st second	−0.00 (0.01) [−0.01; −0.00]	−0.00 (0.02) [−0.00; 0.00]	−0.00 (0.01) [−0.01; −0.00]	1.28	0.280	0.03
2nd second	−0.01 (0.03) [0.01; 0.00]	0.00 (0.03) [−0.01; 0.01]	−0.01 (0.02) [−0.01; 0.02]	1.26	0.285	0.03
3rd second	0.01 (0.05) [−0.00; 0.03]	0.00 (0.05) [−0.01; 0.02]	0.00 (0.02) [−0.01; 0.01]	1.11	0.331	0.03
4th second	0.03 (0.09) [0.01; 0.06]	0.00 (0.04) [−0.02; 0.01]	0.02 (0.06) [0.00; 0.04]	3.68	0.036	0.08
5th second	0.02 (0.08) [0.00; 0.05]	−0.02 (0.04) [−0.03; −0.01]	0.01 (0.06) [−0.00; 0.08]	5.11	0.011	0.11

*Statistics correspond to ANOVA effects of the stimulus category.*

#### Skin Conductance (SC)

The analysis of the SC measurements also showed a significant interaction between *stimulus category* and *time window*, *F*(8,336) = 5.62, *p* = 0.004, η^2^ = 0.12, no main effect for *stimulus category* was found, *F*(2,84) = 2.22, *p* = 0.119, η^2^ = 0.05, and moderate effect was present for time, *F*(4,168) = 5.34, *p* = 0.007, η^2^ = 0.11^5^. Hence, effects of *stimulus category* were analyzed separately for each *time window* (see Table 3B). No effects can be reported for the first 3 s after stimulus onset (*p* > 0.10), but significant effects of *stimulus category* were found for s4, *F*(2,84) = 3.68, *p* = 0.036, η^2^ = 0.08, and s5, *F*(2,84) = 5.11, *p* = 0.011, η^2^ = 0.11. Pleasant pictures, *p* = [0.049; 0.028], *d* = [0.38; 0.42], as well as unpleasant pictures, *p* = [0.160; 0.028], *d* = [0.30; 0.40], elicited higher SC activity compared to neutral pictures with stronger effects for the fifth second (see also Figure 3D). Elevated SC did not differ between pleasant and unpleasant stimulus material, *p* > 0.10, *d* = [0.13; 0.16].

### FR Arousal Versus SC

Surprisingly, all *stimulus categories* induced more activation measured by FR Arousal, which had the highest activation in response to unpleasant pictures and the lowest activation for pleasant pictures. In contrast to FR Arousal, SC activity increased when viewing emotional arousing pictures and decreased for neutral pictures.

### Correlations of Emotional Indicators

**FIGURE 4:**
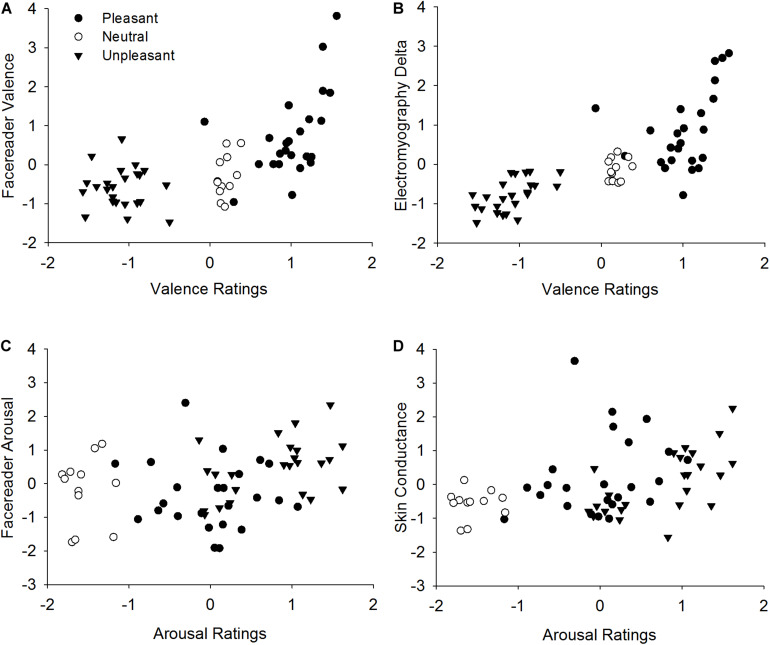
Correlation between valence ratings and Facereader Valence **(A)**, valence ratings and Electromyography Delta **(B)** as well as between arousal ratings and Facereader Arousal **(C)**, arousal ratings and Skin Conductance **(D)**. Values indicate *z*-standardized mean values per stimulus.

In order to provide further information on measurement performance of FR Valence and EMG Delta, correlations between both measures and self-report ratings of emotional valence were calculated. Ratings and measurements of all participants were averaged per stimulus. Self-report valence ratings were highly correlated (see also Table 4) with FR Valence, *r*(58) = 0.63, *p* < 0.001 (see Figure 4A), as well as with EMG Delta activity, *r*(58) = 0.78, *p* < 0.001 (Figure 4B). Visual inspection of the scatterplots revealed a *stimulus category* dependency of the correlations, especially for FR Valence which were highly associated with stimulus ratings of pleasant pictures, *r*(22) = 0.51, *p* = 0.011. However, correlation with unpleasant pictures did not reach significance, *r*(22) = −0.07, *p* = 0.736. In contrast, EMG measurements correlated significantly with valence ratings of pleasant pictures, *r*(22) = 0.41, *p* = 0.044, and unpleasant pictures, *r*(22) = 0.58, *p* = 0.003. This pattern is also reflected by a direct comparison of FR Valence and EMG activity. Overall correlation between these two measures was strong, *r*(58) = 0.80, *p* < 0.001, especially for pleasant stimuli, *r*(22) = 0.80, *p* < 0.001. But the correlation between FR Valence and EMG did not reach significance for unpleasant pictures, *r*(22) = 0.18, *p* = 0.392. These results show that FR Valence is a sensitive indicator for emotional valence and corresponds highly with EMG activity patterns regarding pleasant stimuli. However, it did not predict reactions toward unpleasant emotional content.

**TABLE 4:** Pearson correlations between valence and arousal self-report ratings and all measurements averaged per stimulus (95% confidence intervals in square brackets).

	EMG Delta	SC	FR Valence	FR Arousal
Valence ratings	0.78*** [0.68; 0.86]	−0.13 [−0.36; 0.10]	0.63*** [0.50; 0.74]	−0.48*** [−0.64; −0.29]
Arousal ratings	−0.27* [−0.46; −0.08]	0.40** [0.20; 0.60]	−0.02 [−0.20; 0.16]	0.27* [0.04; 0.48]

*p≤0.05, **p≤0.01, ***p≤0.001, EMG, electromyography; FR, Facereader; SC, skin conductance.

Regarding self-report arousal ratings, FR Arousal measures only correlated weakly, *r*(58) = 0.27, *p* = 0.035 (see Figure 4C), while SC activity showed a moderate relationship, *r*(58) = 0.40, *p* = 0.002 (see Figure 4D and Table 4). Correspondingly, FR Arousal and SC were associated moderately, *r*(58) = 0.33, *p* = 0.009. Regarding measurement specificity, SC activity was uncorrelated, *r*(57) = −0.13, *p* = 0.328, but FR Arousal was highly significantly and negatively related with self-report valence ratings, *r*(57) = −0.42, *p* = 0.001. Thus, unpleasant ratings were associated with higher FR Arousal activity. This demonstrates that FR Arousal as an activity parameter is more predictive in terms of valence than arousal ratings, whereas SC activity is a sensitive and specific indicator of emotional arousal.

As exploratory analyses we compared stimuli with different content by averaging the *z*-scores for each measure (Valence Ratings, FR Valence, EMG Delta (Zygomaticus – Corrugator), Arousal Ratings, FR Arousal, SC) separately for each stimulus group (see Supplementary Material B).

## Discussion

This is the first systematic evaluation of a state-of-the-art AFC software (i.e., FR) to classify facial expressions elicited by standardized emotional pictures in comparison to simultaneously recorded established psychophysiological measures (EMG and SC). We identified great potential for its use as a research tool, with some noteworthy limitations.

### Automatic Facial Coding Versus Psycho-Physiological Research Tools

For pleasant stimuli, FR Valence correlated highly with facial reaction measured by EMG and with valence ratings. Pleasant facial expressions were measured at an equal level of sensitivity by FR, as opposed to measuring them with EMG. In particular, FR Valence as well as EMG showed the strongest positive response toward animals and babies. In contrast to FR Valence, EMG Delta was also associated with different valence intensities for unpleasant stimulus groups (see Supplementary Material B). Thus, sensitivity of EMG is not limited to any one kind of material. Hence, our results indicate that FR is an appropriate measurement alternative to EMG in the context of pleasant facial expressions but cannot yet replace established psychophysiological measurement tools if an unpleasant reactions or arousal processes are measured. FR Valence of pleasant emotion has already been shown to be a very sensitive and specific measure in the case of intense prototypical facial expressions (e.g., Bijlstra and Dotsch, 2011; Lewinski et al., 2014). This can now be generalized to naturally occurring facial responses to pleasant emotional images.

The main advantage of AFC in comparison to other measures is that it does not require direct physical contact and is thus less invasive than physiological indicators. As aforementioned, AFC measurement of emotional expression is even less time consuming because no preparation is needed. This may be an advantage especially for clinical populations in which electrode placement could lead to additional stress for patient groups (e.g., patients with social phobias or autism). In addition, AFC technology can easily be integrated in online research projects through cameras. Therefore, it may replace human coding and psychophysiological measurement in specific research settings.

FR Valence measures were not able differentiate between neutral and unpleasant facial expressions, while EMG was highly sensitive to these differences. Both categories of stimuli led to a negative shift of FR Valence signals, which can either be interpreted as correctly detected unpleasantness while viewing unpleasant pictures but with a negative bias for neutral pictures, or as insufficient sensitivity of AFC in detecting unpleasant facial responses. The latter explanation is more convincing, as it is known that participants show a slightly unpleasant facial reaction toward neutral IAPS pictures (Lang et al., 1993), which is also reflected in the present study. This corresponds with the findings that AFC based on EMFACS shows a worse performance for the detection of unpleasant compared to pleasant facial expressions (Bijlstra and Dotsch, 2011; Lewinski et al., 2014), which might be even more pronounced if participants show emotional expression spontaneously instead of using standardized facial picture inventories (Stöckli et al., 2018).

Another explanation for the lowered sensitivity of unpleasant facial expressions for FR is that EMFACS-based coding of anger, sadness, disgust and fear does not reflect spontaneous unpleasant facial response. In fact, classical basic emotion categories have theoretical and practical shortcomings (Campos et al., 2013; Calvo et al., 2014), and thus, addresses only prototypical facial expressions of basic emotions. Previous work has suggested that AFC is less successful in identifying naturally occurring facial responses (Mortillaro et al., 2015). Hence, future generations of AFC systems have to reach two converging goals: on the one hand, AFC must broaden its spectrum of measurable emotional categories to encompass naturalistic emotional complexity. On the other hand, AFC can potentially be improved if deep learning algorithms are not based on prototypical facial expressions of basic emotions, but on naturalistic facial responses to pleasant and unpleasant emotional situations.

Measures of overall facial movement (FR Arousal) were even less associated to participants’ arousal ratings, while SC correlated with self-reported arousal. In line with previous findings, SC increased in the present study during the presentation of emotional stimuli unspecifically regarding emotional valence (Kreibig, 2010; Siegel et al., 2018). Due to higher muscular complexity during unpleasant compared to pleasant states, FR Arousal rather corresponds with emotional valence than arousal ratings. While FR Valence did not show a significant correlation with valence ratings of unpleasant pictures, FR Arousal showed at least a marginally significant enhancement for unpleasant compared to neutral or pleasant pictures. Future research has to investigate whether a combination of both FR parameters can improve valence measurement sensitivity, especially for unpleasant facial responses. It is even possible that avoidance responses toward unpleasant stimuli, like turning the head slightly away or other head movements, might indicate such avoidance behaviors and hence, could be a potential alternative in detection unpleasant responses via AFC. Other contactless alternatives to record emotional arousal such as indirect heart rate measurement with video-based photoplethysmography (Tasli et al., 2014), thermal variations of the face (Kosonogov et al., 2017), pupillometry (Höfle et al., 2008), or speech analysis (Cowie et al., 2001; Pantic and Rothkrantz, 2003), should be explored in more detail.

Differences in latencies between EMG and FR are also a critical issue. EMG signals already differentiated strongly between *stimulus categories* immediately after stimulus onset, whereas FR measurements showed an unexpected latency of 2 s. This delay of FR will possibly be improved with progression in computer science. However, for practical use of FR as a research tool, this is problematic. In most settings, emotional responses change quickly and often researchers will not have inter-trial intervals as long as those in this study. Especially for highly dynamic stimulus material, such as emotionally complex video material, this measurement delay can potentially lead to a misattribution of emotional reactions and the corresponding emotion eliciting scene. In contrast to FR, facial response measured by EMG is most clear cut during the first second (Dimberg et al., 2000) and is already modulated about 100 ms after stimulus onset (Künecke et al., 2014), which demonstrates the close link between facial muscle activity and automatic affective evaluation.

### Limitations and Perspectives

Several limitations of the study need to be addressed. Because all measures were recorded simultaneously, the question remains as to whether the EMG electrodes might interfere with FR measurements. EMG electrodes were, of course, located above the corrugator. However, FR measures activity of the corrugator mostly depend on activity of AU 4 (Brow Lowerer), which is not covered by the electrodes in our study. Most importantly, the electrodes do not interfere with movements of the brow or the cheek. Moreover, naturally occurring static features of the face such as birthmarks, moles, or piercings should not interfere with FR measurements if it were to qualify for an ecologically valid measurement.

Aggregating data in 1-s bins is rather coarse considering the dynamic of facial expressions (e.g., Matsumoto and Hwang, 2018). In addition, advanced analysis methods (e.g., peak-detection algorithms) are not implemented for FR measurements yet, so we decided to follow the analysis rationale of FR also in the analysis of EMG and SC. Only this enabled a fair comparison between the different measurements. However, for the analysis of SC this might be a disadvantage, because SC is sometimes reported as means of peak activation (SCR). In comparison to previous finding (e.g., Lang et al., 1993; Bradley et al., 2001, 2008), effect size for SC appear to be smaller. Hence, our effect sizes may mark a lower bound for SC effects. However, our results are statistically significant and showed a typical pattern of increased SC for erotica, attack and mutilation scenes and are therefore in line with previous findings (e.g., Bradley et al., 2001).

In order to further establish AFC and FR in particular as a measurement tool, future research should investigate specific measurement impairments of different unpleasant emotions and the influence of emotional intensity of different stimulus material. The most convincing explanation for the present findings is a limited sensitivity of FR for unpleasant facial expressions compared to pleasant expressions. However, the IAPS pictures used for emotion induction were not selected systematically to elicit distinct unpleasant emotions. Therefore, further studies should investigate possible differences in measurement sensitivity of FR regarding distinct unpleasant emotions. For example, categorical accuracies of standardized picture inventories suggest that performance might be better for disgusted or sad facial expressions (Bijlstra and Dotsch, 2011; Lewinski et al., 2014). But these preliminary results need to be expanded by collecting naturally occurring emotional responses (Zhang et al., 2014).

Furthermore, it is rather unclear whether different emotional intensity levels of stimuli types influence FR measurement performance. As an alternative to emotional scenes, pictures of emotional facial expressions of others can be useful for emotion induction; emotional scenes and faces can elicit a different psychophysiological response (e.g., Alpers et al., 2011) but similar activation of the facial muscles (Eisenbarth et al., 2011). Processing emotional facial expressions of others demands these two distinct processes. Viewing such pictures elicits emotion and triggers automatic affective evaluative reactions associated with corresponding facial response (Neumann et al., 2014). Simultaneously, emotional facial expressions perceived in others initiates motor-mimicry processes (e.g., Rizzolatti and Craighero, 2004), which are at the foundation of a broad variety of social-psychological phenomena like empathy (e.g., Gallese, 2001). Using portrait pictures of facial expressions instead of emotional scenes could show whether FR is capable of automatic emotional reactions. The results could then be broadened to apply to more naturalistic emotional stimulus material.

Because our sample consisted mostly of young European participants, further replications with more diverse samples are needed to document generalizability. We expect that EMG is very robust but FR may well be affected by tone of skin and facial shape. Generalizability to naturally occurring situations is also limited because the videos for the FR analyses were recorded under optimal conditions (e.g., seated participants in a laboratory setting, optimal lighting, well controlled environment). However, for a research tool creating such conditions may not be too demanding. Together with other computer-based methods of scoring of emotional sentiments such as text analysis (e.g., Alpers et al., 2005), there may be a wide array of applications for AFC in general. However, AFC is a fast-developing field and ethical application of this technology needs to ensured.

## Conclusion

The present study indicates that FR is a promising new research tool. At its present state, such software provides an acceptable alternative to EMG for research concerning pleasant facial responses when the timing of the response onset is not critical. However, the software tested here was neither able to differentiate between unpleasant and neutral responses, nor indicate the extent of emotional arousal expressed by our participants. In contrast to FR, well-established physiological measures of facial muscle response and the activity of sweat glands indicated valence and arousal reactions with improved sensitivity and specificity. This novel technology has yet to make strides to surpass the sensitivity of long-established methods, but it is a promising new measurement alternative for research settings that require non-contact assessment of emotional responses.

## Data Availability Statement

Data for the study are available at https://madata.bib.uni-mannheim.de/id/eprint/319.

## Ethics Statement

The study involving human participants was reviewed and approved by EK Mannheim 09-1/2018. The participants provided their written informed consent to participate in this study. Written informed consent was obtained from the individual(s) for the publication of any potentially identifiable images or data included in this article.

## Author Contributions

TH, GA, and AG conceived and designed the study. TH conducted the data acquisition, analysis, and interpretation of the results, and also drafted the work. GA, AG, and UF contributed to the interpretation of the results and writing of the manuscript.

## Conflict of Interest

The authors declare that the research was conducted in the absence of any commercial or financial relationships that could be construed as a potential conflict of interest.

## Footnotes

1 The required sample size was determined *a priori* using G^∗^Power 3.1 (Faul et al., 2007). A sample size of 43 participants is required to detect moderate ANOVA effects with a maximum alpha and beta error of 5% regarding the factor *stimulus category*.

2 IAPS picture numbers: 1604, 1630, 1812, 2141, 2152, 2165, 2205, 2250, 2301, 2383, 2396, 2514, 2683, 2850, 2900, 3010, 3051, 3150, 3213, 3400, 3530, 4180, 4250, 4290, 4490, 4505, 4525, 4659, 4660, 4668, 4680, 4690, 4695, 5199, 5780, 5814, 5829, 5831, 5870, 6313, 6350, 6550, 7002, 7009, 7010, 7020, 7035, 7041, 7493, 7550, 9000, 9220, 9280, 9290, 9295, 9330, 9342, 9405, 9830, 9921.

3 The mean activation of all AUs (with inversed AU43 – eyes closed) are aggregated over the past 60 s (PA) and for the current AU activation (CA). Both averages are subtracted from each other to receive Corrected Activation Values [*CAV* = *Max*(*0, CA – PA*)] in order to correct for AUs that are always activated and therefore might enhance an individual bias. Finally, FR Arousal is the average of the five highest active CAVs [*FR Arousal* = *Mean* (*5 max values of CAV*)].

4 *Post hoc* calculation showed no *gender* specific differences for valence ratings of pleasant pictures, *t*(41) = 0.91, *p* = 0.366, *d* = 0.29, or neutral pictures, *t*(41) = 0.43, *p* = 0.671, *d* = 0.11, but for unpleasant pictures, *t*(41) = 2.05, *p* = 0.047, *d* = 0.66. Females, *M* = 7.64, *SD* = 0.71, rated unpleasant pictures more unpleasant than males, *M* = 7.19, *SD* = 0.65, which can be traced back to a higher sensitivity of females toward unpleasant emotional stimuli (Lang et al., 1998). However, *post hoc* calculation showed no *gender* specific differences for arousal ratings of pleasant pictures, *t*(41) = 1.48, *p* = 0.146, *d* = 0.47, neutral pictures, *t*(41) = 0.36, *p* = 0.720, *d* = 0.11, or unpleasant pictures, *t*(41) = 1.07, *p* = 0.291, *d* = 0.34.

5 All of the analyses were also conducted with the factor *gender*, but no interaction or main effect can be reported (*p* > 0.10).
