# Supplementary material for: Read My Face: Automatic Facial Coding Versus Psychophysiological Indicators of Emotional Valence and Arousal
Source: Front Psychol. 2020 Jun 19;11:1388. doi: 10.3389/fpsyg.2020.01388 (PMC7316962; doi:10.3389/fpsyg.2020.01388)
Supplement: Supplementary file 1 [file Table_1.pdf]

# *Supplementary Material*

## A) Analysis of Action Unit (AU) 4/12 and Corrugator/Zygomaticus EMG Activities

*Facereader*[1]: The ANOVA for AU4 (Brow Lowerer, Frowning) activity extracted by FR, showed a significant interaction effect between *stimulus category* and *time window*, $F(8, 336) = 3.33$, $p = .020$, $\eta^2 = .07$, a significant main effect for *stimulus category*, $F(2, 84) = 4.63$, $p = .016$, $\eta^2 = .10$, and a strong main effect for *time window*, $F(4, 168) = 9.44$, $p < .001$, $\eta^2 = .18$. Hence, effects of *stimulus category* were analyzed separately for each time point (see Table A1). *Stimulus categories* modulated AU4 activities significantly after second two (see Figure A1).

The ANOVA for AU12 (Lip Corner Pull, Smiling) showed a significant interaction effect between *stimulus category* and *time window*, $F(8, 336) = 4.30$, $p = .035$, $\eta^2 = .09$, a marginal significant main effect for *stimulus category*, $F(2, 84) = 2.90$, $p = .087$, $\eta^2 = .06$, and a significant main effect for *time windows*, $F(4, 168) = 6.24$, $p = .012$, $\eta^2 = .13$. Hence, effects of *stimulus category* were analyzed separately for each time point (see Table A1). *Stimulus categories* modulated AU12 activities significantly only for the fifth second (see Figure A2).

*Electromyography (EMG)*: The ANOVA for EMG Corrugator showed a strong interaction between *stimulus category* and *time window*, $F(8, 336) = 5.79$, $p = .001$, $\eta^2 = .12$, a strong main effect for *stimulus category*, $F(2, 84) = 17.03$, $p < .001$, $\eta^2 = .29$, and no significant effect for *time windows*, $F(4, 168) = 1.65$, $p = .206$, $\eta^2 = .04$. Hence, effects of *stimulus category* were analyzed separately for each time point (see Table A2). *Stimulus categories* strongly modulated EMG Corrugator activities within each *time window* (see Figure A3).

The ANOVA for EMG Zygomaticus revealed a marginal significant interaction between *stimulus category* and *time window*, $F(8, 336) = 2.76$, $p = .070$, $\eta^2 = .06$, a main effect for *stimulus category*, $F(2, 84) = 3.79$, $p = .050$, $\eta^2 = .08$, and *time windows*, $F(4, 168) = 4.91$, $p = .016$, $\eta^2 = .11$. Hence, effects of *stimulus category* were analyzed separately for each time point (see Table A2). *Stimulus categories* modulated EMG Zygomaticus activities only between second and fourth second after stimulus onset (see Figure A4).

Table A1

*Mean automatic facial coding activity of Action Unit 4 (Brow Lowerer) and 12 (Lip Corner Pull; standard deviations in parenthesis, 95% confidence intervals in square brackets, difference to baseline in arbitrary units [AU]), separately for time windows and stimulus categories. Statistics correspond to ANOVA effects of the stimulus category.*

| | Action Unit 4 (Brow Lowerer) [AU] | | | | | |
|---|---|---|---|---|---|---|
| | Pleasant | Neutral | Unpleasant | $F(2, 84)$ | $p$-value | $\eta^2$ |
| 1st second | 0.00 (1.30)<br>[-0.40; 0.35] | 0.20 (2.08)<br>[-0.43; 0.80] | 0.21 (1.53)<br>[-0.26; 0.63] | 0.40 | .661 | .01 |
| 2nd second | 0.49 (2.74)<br>[-0.35; 1.26] | 1.35 (2.33)<br>[0.67; 2.04] | 1.29 (2.95)<br>[0.41; 2.17] | 2.50 | .090 | .06 |
| 3rd second | -0.40 (3.90)<br>[-1.58; 0.72] | 1.49 (3.92)<br>[0.35; 2.71] | 1.61 (3.69)<br>[0.56; 2.78] | 5.97 | .005 | .13 |
| 4th second | -0.96 (4.23)<br>[-2.35; 0.25] | 0.84 (4.45)<br>[-0.39; 2.26] | 1.31 (3.68)<br>[0.25; 2.45] | 4.82 | .013 | .10 |
| 5th second | -2.21 (4.68)<br>[-3.73; -0.90] | -0.58 (4.66)<br>[-1.94; 0.86] | 0.15 (3.93)<br>[-1.01; 1.36] | 3.62 | .037 | .08 |
| | Action Unit 12 (Lip Corner Pull) [AU] | | | | | |
| | Pleasant | Neutral | Unpleasant | $F(2, 84)$ | $p$-value | $\eta^2$ |
| 1st second | -0.05 (0.42)<br>[-0.19; 0.06] | -0.03 (0.12)<br>[-0.07; 0.00] | -0.03 (0.31)<br>[-0.14; 0.05] | 0.08 | .905 | .00 |
| 2nd second | -0.12 (0.55)<br>[-0.30; 0.01] | -0.03 (0.14)<br>[-0.08; 0.00] | -0.08 (0.41)<br>[-0.21; 0.03] | 0.66 | .505 | .02 |
| 3rd second | 0.20 (0.92)<br>[-0.07; 0.49] | 0.03 (0.33)<br>[-0.05; 0.14] | -0.04 (0.54)<br>[-0.20; 0.13] | 1.45 | .241 | .03 |
| 4th second | 0.84 (2.57)<br>[0.19; 1.73] | 0.15 (0.82)<br>[-0.04; 0.43] | 0.07 (0.88)<br>[-0.14; 0.37] | 3.24 | .069 | .07 |
| 5th second | 1.04 (3.09)<br>[0.27; 2.11] | 0.00 (0.14)<br>[-0.05; 0.03] | 0.05 (0.68)<br>[-0.13; 0.27] | 4.29 | .041 | .09 |

Table A2

*Mean electromyography (EMG) activity of corrugator and zygomaticus (standard deviations in parenthesis, 95% confidence intervals in square brackets, difference to baseline in microvolts [mV]), separately for time windows and stimulus categories. Statistics correspond to ANOVA effects of the stimulus category.*

| | EMG Corrugator [mV] | | | | | |
|---|---|---|---|---|---|---|
| | Pleasant | Neutral | Unpleasant | $F(2, 84)$ | $p$-value | $\eta^2$ |
| 1st second | -0.26 (1.05)<br>[-0.60; 0.02] | 0.13 (0.68)<br>[-0.08; 0.33] | 0.69 (0.85)<br>[0.44; 0.94] | 23.90 | <.001 | .36 |
| 2nd second | -0.56 (1.68)<br>[-1.08; -0.08] | 0.29 (1.03)<br>[0.01; 0.61] | 1.50 (1.95)<br>[0.93; 2.10] | 18.05 | <.001 | .30 |
| 3rd second | -0.55 (2.09)<br>[-1.13; 0.10] | 0.30 (1.30)<br>[-0.07; 0.71] | 1.31 (1.91)<br>[0.77; 1.92] | 16.08 | <.001 | .28 |
| 4th second | -0.53 (1.98)<br>[-1.07; 0.10] | 0.09 (1.27)<br>[-0.25; 0.48] | 1.04 (1.85)<br>[0.51; 1.63] | 12.39 | <.001 | .23 |
| 5th second | -0.56 (1.57)<br>[-1.01; -0.09] | 0.02 (1.12)<br>[-0.29; 0.36] | 0.67 (1.50)<br>[0.26; 1.15] | 10.23 | <.001 | .20 |
| | EMG Zygomaticus [mV] | | | | | |
| | Pleasant | Neutral | Unpleasant | $F(2, 84)$ | $p$-value | $\eta^2$ |
| 1st second | 0.06 (0.31)<br>[-0.03; 0.16] | -0.04 (0.39)<br>[-0.14; 0.08] | -0.08 (0.22)<br>[-0.16; -0.02] | 2.41 | .099 | .05 |
| 2nd second | 0.98 (2.92)<br>[0.30; 2.00] | 0.10 (0.71)<br>[-0.09; 0.34] | 0.09 (0.50)<br>[-0.05; 0.24] | 4.27 | .041 | .09 |
| 3rd second | 1.27 (3.81)<br>[0.37; 2.58] | 0.16 (1,11)<br>[-0.11; 0.54] | 0.21 (0.64)<br>[0.03; 0.41] | 3.87 | .048 | .08 |
| 4th second | 1.07 (3.16)<br>[0.29; 2.13] | 0.15 (0.75)<br>[-0.05; 0.39] | 0.21 (0.54)<br>[0.06; 0.39] | 3.55 | .060 | .08 |
| 5th second | 0.80 (2.18)<br>[0.25; 1.53] | 0.34 (1.16)<br>[0.05; 0.72] | 0.25 (0.57)<br>[0.09; 0.42] | 1.85 | .175 | .04 |

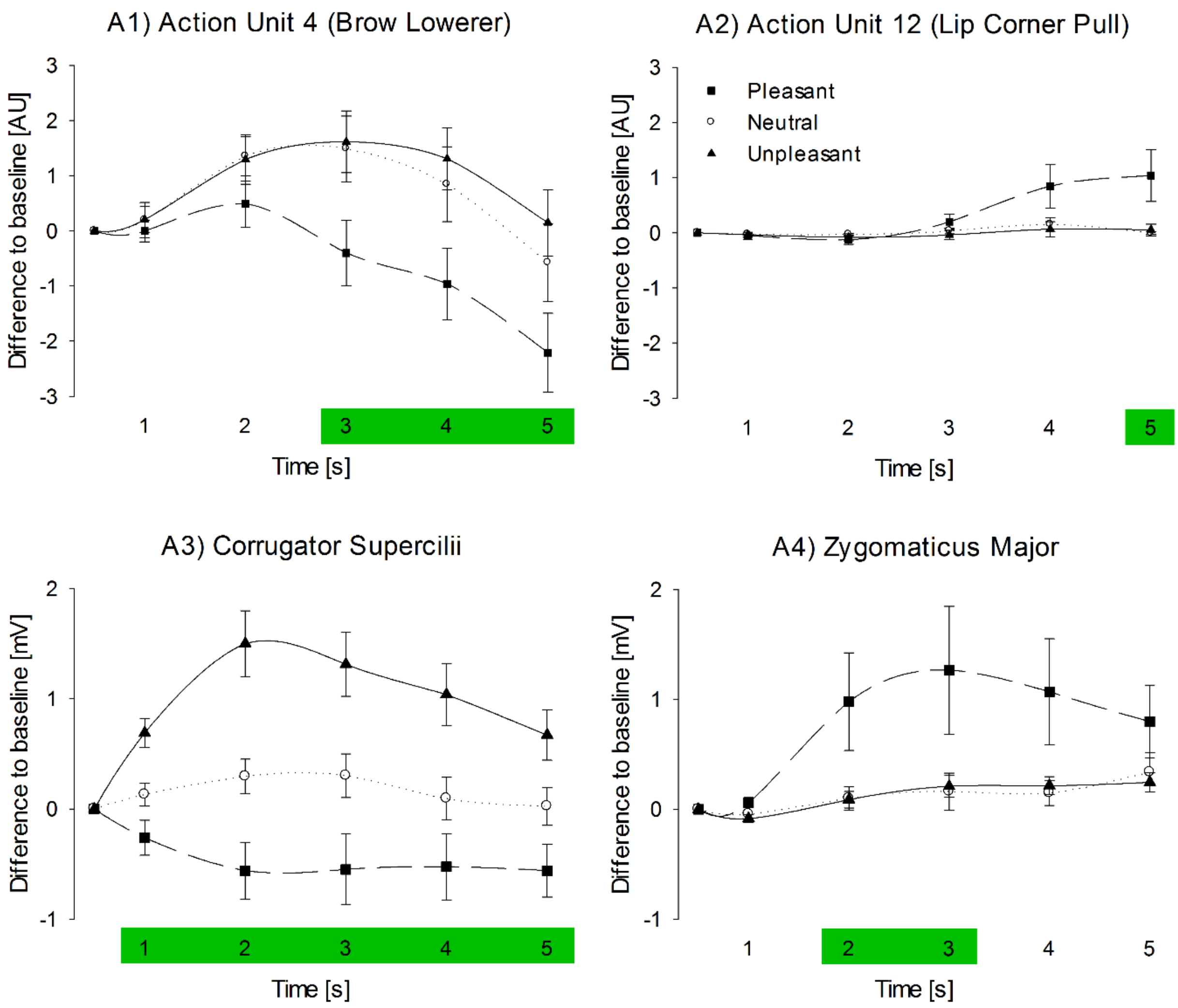


*Figure A*: Averaged activities of automatic facial coding of action unit 4 (Panel A1) and 12 (Panel A2), as well as electromyography (EMG) activities of corrugator (Panel A3) and zygomaticus muscle (Panel A4) measurements (difference to baseline in arbitrary units [AU] and millivolt [mV]), separate for time windows after picture onset and stimulus category. Error bars are standard errors of the mean. Green areas indicate significant differences between stimulus categories.

## B) Exploratory analysis of stimulus groups

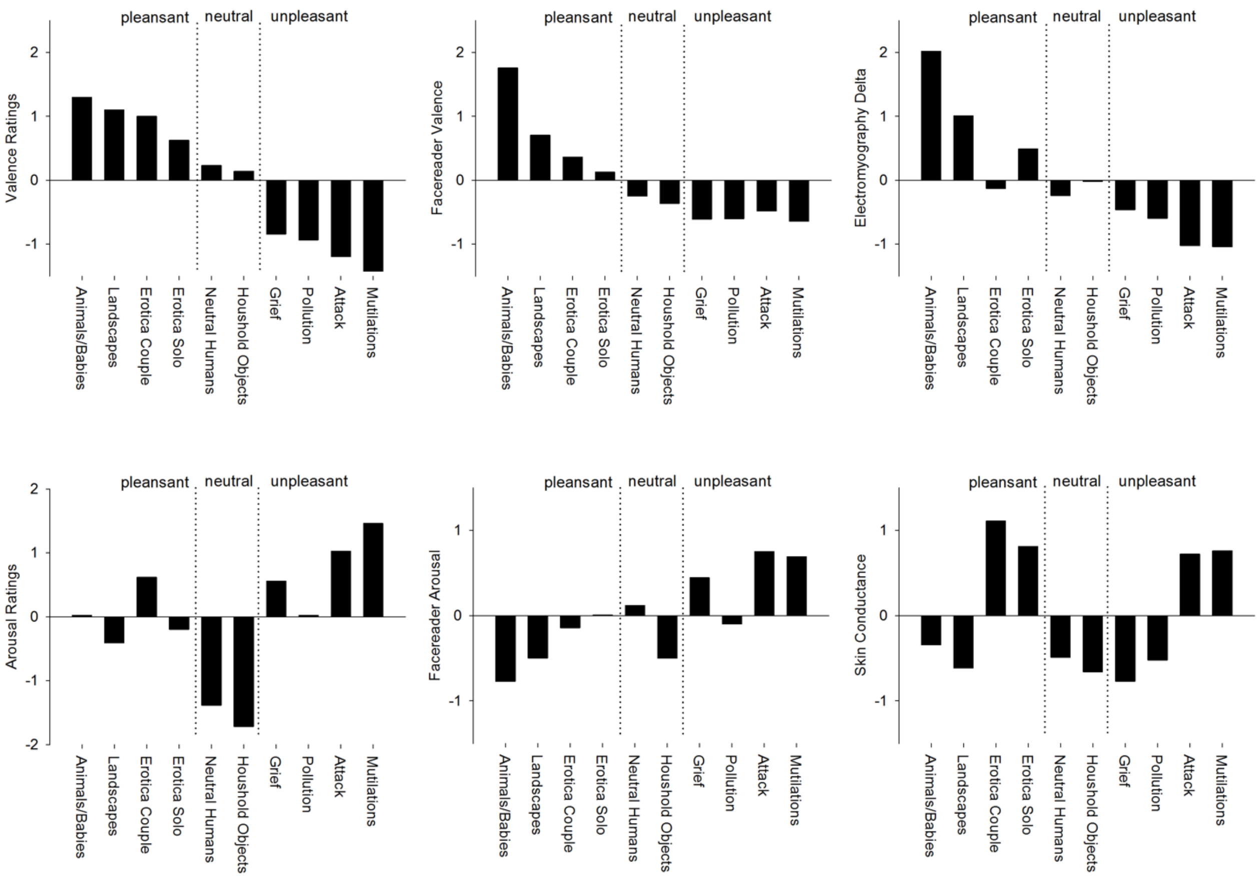


*Figure B*. Values indicate z-standardized mean values per stimulus group separately for all measures (Valence Ratings, Facereader Valence, Electromyography Delta (Zygomaticus – Corrugator), Arousal Ratings, Facereader Arousal, Skin Conductance). Pleasant stimuli consist of animals or babies, landscapes, erotica couples and erotica solo, neutral stimuli consist of neutral humans and household objects, and unpleasant stimuli consist of grief, pollution, attack, and mutilation scenes. Facereader Valence and EMG Delta were modulated by different valence intensities for pleasant stimulus groups. In contrast to Facereader Valence, EMG Delta was also associated with different valence intensities for unpleasant stimulus groups. With regards of arousal measures, SC was increased erotica, attack and mutilation scenes, whereas Facereader Arousal only showed increased values for highly arousing unpleasant stimulus groups.

## Footnotes

[1] Action Units were analyzed with Version 7.1 of the Facereader Software (Noldus Information Technology)
